# LIMK Regulates Tumor-Cell Invasion and Matrix Degradation Through Tyrosine Phosphorylation of MT1-MMP

**DOI:** 10.1038/srep24925

**Published:** 2016-04-27

**Authors:** Emilie Lagoutte, Clémentine Villeneuve, Laurence Lafanechère, Claire M. Wells, Gareth E. Jones, Philippe Chavrier, Carine Rossé

**Affiliations:** 1 Institut Curie, PSL Research University, CNRS UMR 144, Membrane and Cytoskeleton Dynamics, 75248 cedex 05, Paris, France; 2 Univ. Grenoble Alpes, INSERM U823, Institut Albert Bonniot, CRI, Team 3 “Polarity, Development and Cancer”, F-38000 Grenoble France; 3 Division of Cancer Studies, King’s College London, London, United Kingdom; 4 Randall Division of Cell and Molecular Biophysics, King’s College London, London, United Kingdom

## Abstract

During their metastatic spread, cancer cells need to remodel the extracellular matrix in order to migrate through stromal compartments adjacent to the primary tumor. Dissemination of breast carcinoma cells is mediated by membrane type 1-matrix metalloproteinase (MT1-MMP/MMP14), the main invadopodial matrix degradative component. Here, we identify MT1-MMP as a novel interacting partner of dual-specificity LIM Kinase-1 and -2 (LIMK1/2), and provide several evidence for phosphorylation of tyrosine Y573 in the cytoplasmic domain of MT1-MMP by LIMK. Phosphorylation of Y573 influences association of F-actin binding protein cortactin to MT1-MMP-positive endosomes and invadopodia formation and matrix degradation. Moreover, we show that LIMK1 regulates cortactin association to MT1-MMP-positive endosomes, while LIMK2 controls invadopodia-associated cortactin. In turn, LIMK1 and LIMK2 are required for MT1-MMP-dependent matrix degradation and cell invasion in a three-dimensional type I collagen environment. This novel link between LIMK1/2 and MT1-MMP may have important consequences for therapeutic control of breast cancer cell invasion.

Tumor cell motility is required for local invasion and dissemination of cancer cells from the primary tumor^1^. Proteolytic degradation of the extracellular matrix (ECM) is one intrinsic property of metastatic tumor cells allowing transmigration through the basal membrane and invasion through the stroma mainly composed of type I collagen. Remodeling of ECM by cancer cells depends on matrix-degrading proteases, including matrix metalloproteinases (MMPs)^2^,^3^. Membrane-anchored Type MT1-MMP, also termed MMP14, has been recognized as a major protease involved in dissemination of carcinoma cells and during cancer progression^4^,^5^,^6^,^7^,^8^,^9^^,^^52^.

MT1-MMP is up-regulated in human cancers, including in breast cancers and is enriched at the front of invasive lesions^8^,^9^,^10^,^11^,^12^. In breast adenocarcinoma-derived cell lines such as MDA-MB-231 and BT-549, a significant fraction of MT1-MMP is internalized from the cell surface^13^,^14^ and accumulates in VAMP7-, Rab7-positive late endosomes/lysosomes from where it can recycle to specific matrix-degradative actin-based plasma membrane domains called invadopodia^15^,^16^,^17^,^18^,^19^,^20^,^21^,^22^. Invadopodia formation and function in pericellular matrix degradation requires assembly of two F-actin/cortactin pools. One pool depends on the concerted activity of an N-WASP-Arp2/3 complex, cofilin and cortactin, leading to the assembly of an invadopodial F-actin core on the cytoplasmic face of the plasma membrane in contact with the matrix. Functions for invadopodial F-actin include driving plasma membrane protrusion^23^,^24^,^25^,^26^, and stabilizing MT1-MMP at the cell surface via direct interaction with its cytoplasmic tail^27^. A second F-actin/cortactin pool is found as puncta on the cytosolic face of MT1-MMP, Rab7-positive endosomes and requires endosomal WASH complex, which is necessary for MT1-MMP delivery at invadopodia^19^,^22^,^28^.

The LIM kinase family comprises two related protein kinases (LIMK1 and LIMK2) with dual-specificity serine/threonine and tyrosine activity^29^,^30^,^31^. The major LIMK substrates identified so far are cofilin-family members^29^. Phosphorylation of cofilin on Serine residue 3 by LIMKs inhibits its actin severing activity and thus has a major influence on actin cytoskeleton organization^29^ including on invadopodial actin dynamics^32^,^33^,^34^,^35^. It is postulated that LIMKs are required for cell invasion by promoting formation of an invasive path by cancer cells in a 3D type I collagen environment during collective migration^36^,^37^,^38^. In addition to the carboxy-terminal kinase domain, LIMKs possess protein-protein interaction domains including two amino-terminal LIM domains and a central PDZ domain^29^. Interestingly, the cytoplasmic domain of MT1-MMP with critical trafficking and localization regulatory functions^22^,^39^ contains three potential phospho-residues (T567, Y573 and S577). Phosphorylation of Y573 by Src-kinase regulates tumor cell migration and affects tumor progression through an unknown mechanism^40^,^41^. In addition, the MT1-MMP cytoplasmic domain contains a carboxy-terminal “DKV” motif^42^.

In this study, we provide evidence that MT1-MMP and LIMK interact through the MT1-MMP “DKV” cytoplasmic motif and that Y573 can be phosphorylated by LIMK1. We find that late endocytic MT1-MMP promotes cortactin accumulation in endosomal puncta; endosomal cortactin accumulation is further enhanced by overexpression of MT1-MMP mutant with a Y573E substitution mimicking MT1-MMP phosphorylation by LIMK whilst a non-phosphorylatable Y573F variant has the opposite effect on cortactin. Reciprocally, consistent with its association with MT1-MMP-positive endosomes, LIMK1 is required for endosomal accumulation of cortactin. On the contrary, silencing of LIMK2 does not affect endosomal cortactin. Finally, we find that both LIMK1 and LIMK2 are required for invadopodia formation, MT1-MMP-dependent matrix degradation and invasive migration through 3D collagen. Taken together, these data suggest non-redundant functions of the two LIMK isoforms during MT1-MMP-dependent breast tumor cell invasion.

## Material & Methods

### Antibodies

Anti-cortactin (Clone 4F11), rabbit anti-p34Arc, anti-phospho-tyrosine (Clone 4G10), rabbit anti-TKS5 and mouse anti-MT1-MMP monoclonal antibodies were obtained from Millipore. Monoclonal anti-p34Arc was purchased from Synaptic System. Rabbit anti-Rab7, rabbit anti-cofilin, rabbit anti-phospho-cofilin, rabbit anti-LIMK1 and rabbit anti-LIMK2 antibodies were purchased from Cell Signaling. Rabbit anti-GADPH antibodies were purchased from Santa Cruz. AlexaFluor–phalloidin was from Invitrogen. Horseradish peroxidase-conjugated and fluorescently conjugated secondary antibodies were from Jackson ImmunoResearch Laboratories.

### Plasmid constructs

MT1-MMPmCherry (MT1-MMPmCh) with the tag inserted in the extracellular domain to minimize interference with cytoplasmic domain trafficking motifs has been reported previously^43^. Plasmid construct expressing DsRed-tagged rat cortactin was the kind gift of Dr M.A. McNiven (Mayo Clinic, Rochester, MI, USA). Expression plasmids encoding GFP tagged LIMK1, and LIMK2 were generated using GatewayTM Technology (Invitrogen) and all plasmids were sequenced^44^.

Mutants of MT1-MMP were made by site-directed mutagenesis of MT1-MMPmCh using the QuikChange Kit from Stratagene with the following oligonucleotides: MT1-MMPmChΔCter, 5′-ttcagacgccatgggtgacccaggcgactgctc-3′(sense) and 5′-gagcagtcgcctgggtcagggatggcgtctgaa–3′ (anti-sense); MT1-MMPmChΔDKV, 5′-tgccagcgttccctgctgtgaaaggtctgatctagaggg-3′(sense) and 5′-ccctctagatcagacctttcacagcagggaacgctggca-3′ (anti-sense); MT1-MMPmChY573F, 5′-aggcgactgctcttctgccagcgttcc-3′ (sense) and 5′-ggaacgctggcagaagagcagtcgcct-3′ (anti-sense); MT1-MMPmChY573E, 5′-caggcgactgctcgagtgccagcgttccc-3″ (sense) and 5′-gggaacgctggcactcgagcagtcgcctg-3′ (anti-sense).

### Cell culture, transfection, stable cell lines and gene silencing

The human breast adenocarcinoma cell line MDA-MB-231 (American Type Culture Collection, ATCC HTB-26) was maintained in L-15 culture medium (Sigma-Aldrich) with 2 mM glutamine and 15% FCS at 37 °C in 1% CO_2_. MDA-MB-231 cells stably expressing MT1-MMPmCh were cultured in the presence of 0.5 mg/mL G418^43^. MDA-MB-231 cells were transfected with plasmid constructs by using Amaxa nucleotransfection. Cells were analyzed 24 h after transfection. Small inhibitory RNAs targeting MT1-MMP (MMP14, L-004145-00) were SMARTpoolON-TARGETplus from Dharmacon. Cortactin (CTTN, SI02661960 and SI02662485), LIMK1 (LIMK1, SI00605542 and SI00605549) and LIMK2 (LIMK2, SI02665334 and SI02758490) siRNAs were purchased from Qiagen. Cells were treated with specific siRNA (50 nM final concentration) with Lullaby reagent (OZ Biosciences) and analyzed 72 h after treatment.

### Indirect immunofluorescence, image acquisition and analysis

MDA-MB-231 cells were cultured on gelatin-coated coverslips and processed for immunofluorescence microscopy as previously described^24^. Briefly, cells were pre-extracted with 0.5% Triton ×-100 in 4% paraformaldehyde in PBS during 90s and then fixed in 4% paraformaldehyde in PBS for 20 min and stained for immunofluorescence microscopy with cortactin antibodies. Images were acquired with a wide-field Eclipse 90i Upright Microscope (Nikon) using a 100 × Plan Apo VC 1.4 oil immersion objective and a highly sensitive cooled interlined charge-coupled device (CCD) camera (Roper CoolSnap HQ2). As MT1-MMP-positive endosomes are about 0.5 μm to 2 μm diameter, a z-stack of 10–15 images with 0.2 μm interval between consecutive images was chosen in order to avoid overlap of multiple MT1-MMP endosomes. Z-stack of images were taken by mean of a piezoelectric motor (LVDT, Physik Instrument) from the adherent surface of the cells. Then images were deconvoluted^45^, and a maximum intensity projection (MIP) was performed with ImageJ software. For detection of cortactin on MT1-MMP-containing endosomes, a CellProfiler pipeline was constructed^46^; first, MIP of deconvoluted images of MT1-MMPmCh and cortactin was computed; then cortactin spots were identified using a Laplacian of Gaussian filter followed by a watershed on the automatically thresholded MIP image; the MT1-MMPmCh-containing endosomes were identified by thresholding and intensity-based watershed; the number of cortactin spots was identified then checked in a 3-pixels neighborhood around each MT1-MMPmCh vesicle^19^.

### Fluorescent gelatin degradation assay, quantification of pericellular collagenolysis and invasion assays

Fluorescent gelatin degradation assays were performed and quantified as previously described^43^. For degradation assay performed with MDA-MB-231 cells silenced for LIMK, cells were plated for 5 h on fluorescent-gelatin and then were pre-extracted with 0.5% Triton X-100 in 4% paraformaldehyde in PBS during 90 s and then fixed in 4% paraformaldehyde in PBS for 20 min. For degradation assay performed with MT1-MMPmCh-overexpressing cells (WT or mutant), after 3 h on a gelatin substratum more resistant to degradation (see^43^), cells were fixed in 4% paraformaldehyde in PBS for 20 min. Approximately 200 cells from at least three independent experiments were analyzed for each condition. Assays to measure the invasion of cells from multicellular spheroids into native type I collagen assays were performed as described previously^47^. For collagenolysis assays, cells treated with DMSO or Pyr1 (10 μM) or GM6001 were trypsinized and resuspended in 0.2 ml of 2.2 mg/ml collagen I solution (2.5 × 105 cells/ml) loaded on a glass coverslip. After polymerization for 1 h 30 at 37 °C or 20 °C, complete medium was added and collagen-embedded cells were incubated for 24 h at 37 °C in 1% CO2. After fixation in 4% paraformaldehyde in PBS at 37 °C for 30 min, samples were incubated with Col1-3/4C antibodies (2.5 μg/ml) for 2 h at 4 °C, washed extensively with PBS, and counterstained with Cy3-conjugated anti-rabbit IgG antibodies, DAPI, and Alexa Fluor 488–phalloidin to visualize cell shape. Image acquisition was performed with an LSM SP8 NLO confocal microscope (Leica) with a 40× oil objective. Quantification of pericellular collagenolysis was performed and quantified as previously described^22^.

### Linear invadopodia formation assay and quantification

Coverslips were layered with 100 μl of a solution of type I collagen mixed with Alexa Fluor 647–conjugated type I collagen (5% final) at a final concentration of 2.2 mg/ml. After gelling for 3 min at 37 °C, the collagen layer was washed gently in PBS and 1 ml of the cell suspension in L15 medium with 15% FCS (10^5^ cells/ml) was added. Cells were incubated for 45 min at 37 °C in 1% CO_2_ before fixation^22^. Cells were pre-extracted with 0.5% Triton X-100 in 4% paraformaldehyde in PBS during 90 s and then fixed in 4% paraformaldehyde in PBS for 20 min and stained for immunofluorescence microscopy with Tks5 or cortactin antibodies. Images were acquired with a wide-field microscope (Eclipse 90i Upright; Nikon) using a 100× Plan Apo VC 1.4 oil objective and a highly sensitive cooled interlined charge-coupled device (CCD) camera (CoolSnap HQ2; Roper Scientific). A z-dimension series of images was taken every 0.2 μm by means of a piezoelectric motor (Physik Instrumente)^45^. For quantification of Tks5 and cortactin associated with linear invadopodia in cells plated on collagen fibers, three consecutive z-planes corresponding to the plasma membrane in contact with collagen fibers were projected and surface covered by each of the markers was determined using the thresholding command of ImageJ excluding regions <16 pixels (<1 μm) to avoid non-invadopodial structures such as endosomal cortactin-positive patches. Surface covered by each marker was normalized to the total cell surface and values normalized to control cells.

### Immunoprecipitation and kinase assay

For RFP-trap assays, MDA-MB-231 cells overexpressing or not MT1-MMPmCh WT or ΔDKV were lysed in 50 mM Tris-HCl (pH 7.5), 150 mM NaCl, 60 mM beta-glucoside, 10 mM MgCl_2_, 1% Triton X-100, and 10% glycerol with anti-protease and anti-phosphatases. RFP-Trap beads (ChromoTek) were added at concentration recommended by the supplier. For *in vitro* kinase assays, GST-LIMK1 recombinant protein (Abnova) was incubated with precipitated MT1-MMPmCh with RFP-Trap beads (ChromoTek) in stringent conditions (RIPA Buffer: 50 mM Tris-HCl (pH 7.5), 150 mM NaCl, 2 mM EDTA, 10 mM MgCl_2_, 1% NP40, 0.5% Na Deoxycholate, 0.1% SDS) as the substrate in the kinase assay buffer containing 50 mM HEPES, 150 mM NaCl, 5 mM MgCl_2_, 5 mM MnCl_2_, 250 μM ATP for LIMK1. The reaction mix was incubated for 30 min at 30 °C. The reaction was stopped by addition of 2× Laemmli sample buffer. Overexpressed MT1-MMP and phosphorylated bands were respectively determined by SDS-PAGE with an anti-MT1-MMP (Millipore) an anti-phosphotyrosine antibodies (4G10). Immunoprecipitation experiments were replicated at least 3 times except for immunoprecipitation of endogenous MT1-MMP using mouse 2D7 anti-MT1-MMP monoclonal antibody (a kind gift of M.C. Rio and C.L. Tomasetto, IGBMC, Strasbourg, France), which was performed once.

## Results

### LIMKs interact with and phosphorylates the MT1-MMP cytosolic tail

**Figure 1:**
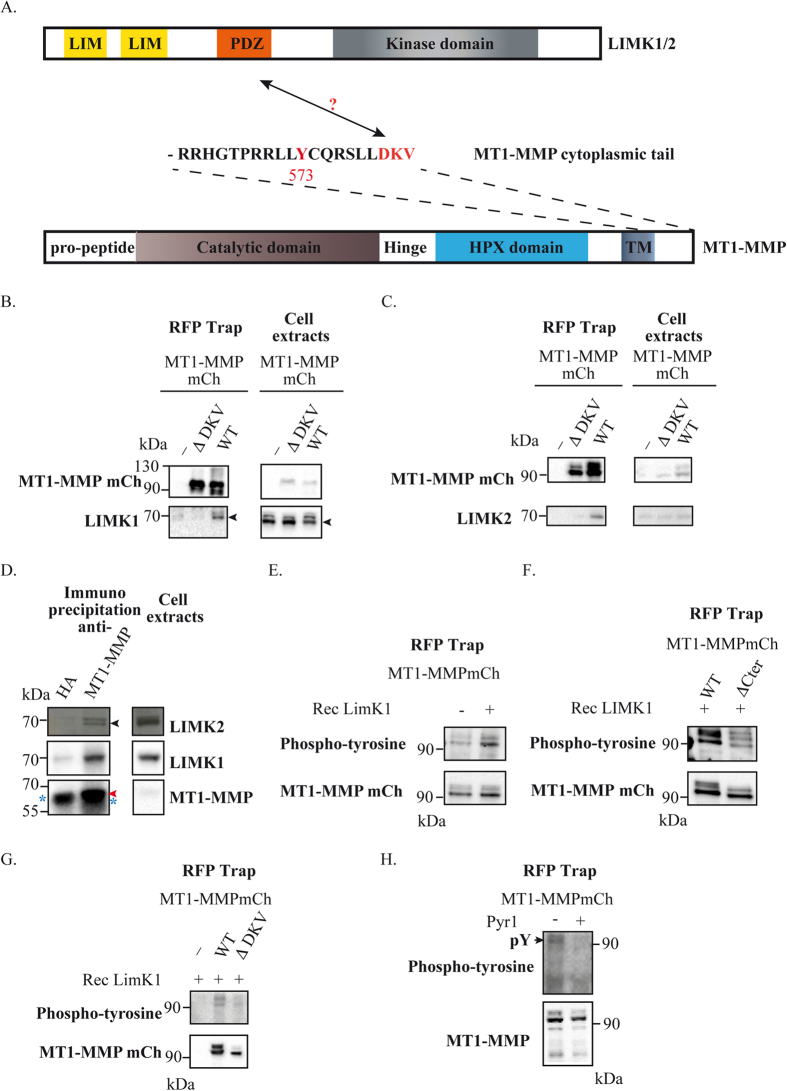
MT1-MMP is a partner and substrate of LIMK1/2. **(A)** Schema of the different domains of MT1-MMP and LIMK **(B,C)** Immunodetection of respectively LIMK1 (**B**) or LIMK2 (**C**) with MT1-MMPmCh WT beads trap in MDA-MB-231 cell lysates. Bound proteins were analyzed by immunoblotting with antibodies against MT1-MMP, LIMK1 and LIMK2. Input lysates (1%) were loaded as control. Arrow indicates specific LIMK1 band. **(D)** Immunodetection of LIMK1 and LIMK2 in anti-MT1-MMP immunoprecipitates in contrast to control mouse IgGs (anti-HA) in MDA-MB-231 cell lysates. Bound proteins were analyzed by immunoblotting with antibodies against MT1-MMP, LIMK1 and LIMK2. Input lysates (1%) were loaded as control. Black arrow indicates LIMK2; red arrow indicates MT1-MMP; blue asterix indicates high chain IgGs. **(E)** Tyrosine phosphorylation by LIMK1 of MT1-MMPmCh WT **(F)** Tyrosine phosphorylation by LIMK1 of MT1-MMPmCh WT and not of MT1-MMPmCh ΔCter beads trap. **(G)** Tyrosine phosphorylation by LIMK1 of MT1-MMPmCh WT beads trap after treatment with DMSO or Pyr1 (20 μM) **(H)** Tyrosine phosphorylation of MT1-MMPmCh WT after treatment with DMSO or Pyr1 (20 μM).

The carboxy-terminal “DKV” motif of MT1-MMP has features of a PDZ domain-binding site^42^ (Fig. 1A). We thus assessed whether LIMKs, which possess a PDZ domain interact with the “DKV” motif of MT1-MMP (Fig. 1A). Wild-type (WT) MT1-MMPmCh or with a truncation of its last three carboxy-terminal residues (ΔDKV) was stably expressed in MDA-MB-231 cells and precipitated using anti-RFP-coupled beads. LIMK1 and LIMK2 could be detected in immunoprecipitates of MT1-MMPmCh WT but not in un-transfected MDA-MB-231 cells or cells expressing truncated ΔDKV MT1-MMP construct (Fig. 1B,C). Interaction of MT1-MMP with Endogenous LIMK1 and LIMK2 were detected in anti-MT1-MMP immunoprecipitates but not with control IgGs (anti-HA) (Fig. 1D).

LIMKs are dual specificity S/T and Y kinases^29^,^30^,^31^. We thus investigated if LIMKs could phosphorylate S, T and/or Y residues present in the cytosolic tail of MT1-MMP (see Fig. 1A). MT1-MMPmCh WT was precipitated under stringent conditions (to avoid potential co-immunoprecipation of associated kinase(s)) and kinase assay was performed in the presence of recombinant LIMK1. Anti-phospho-S or -T antibodies could not detect phosphorylation of T567 or S577 in the cytoplasmic tail of MT1-MMP in the presence of LIMK1 (not shown). However, phospho-S/T antibodies are notoriously difficult to use and thus we cannot completely exclude phosphorylation of these residues by LIMK. We further investigated phosphorylation of MT1-MMP Y573 residue. MT1-MMPmCh WT or deleted of its 20-aa long cytosolic tail (ΔCter) were precipitated and kinase assay was performed in the presence of LIMK1 as above using 4G10 anti-phospho-Y (pY) antibody for detection. Strongest pY signal was observed with MT1-MMPmCh WT in the presence of LIMK1 with several bands corresponding to different MT1-MMP maturation forms (Fig. 1E). Deletion of the cytosolic tail of MT1-MMP (Fig. 1F) lead to basal pY levels possibly due to phosphorylation of tyrosine residue(s) present in the extracellular domain that is(are) not exposed to LIMK under native conditions. Moreover, deletion of the carboxy-terminal DKV motif decreased by 60% the phosphorylation of MT1-MMP by LIMK1 (Fig. 1G), suggesting that interaction of the DKV motif to the PDZ domain of LIMK is required for MT1-MMP phosphorylation. Then, we investigated if MT1-MMP phosphorylation was dependent on the activity of LIMK1/2 in MDA-MB-231 cells. Some pY signal was visible in MT1-MMPmCh immunoprecipitate from MDA-MB-231 cells. Inhibition of LIMK1/2 by Pyr1, a LIMK-specific ATP competitive inhibitor^48^, abolished pY signal associated with MT1-MMPmCh. We conclude that LIMK can phosphorylate MT1-MMP on cytoplasmic Y573 residue and phosphorylation requires the carboxy-terminal DKV motif likely by mediating interaction with LIMK PDZ domain.

### Endosomal cortactin association is regulated by LIMK1 phosphorylation of Y573 on MT1-MMP’s cytosolic tail

**Figure 2:**
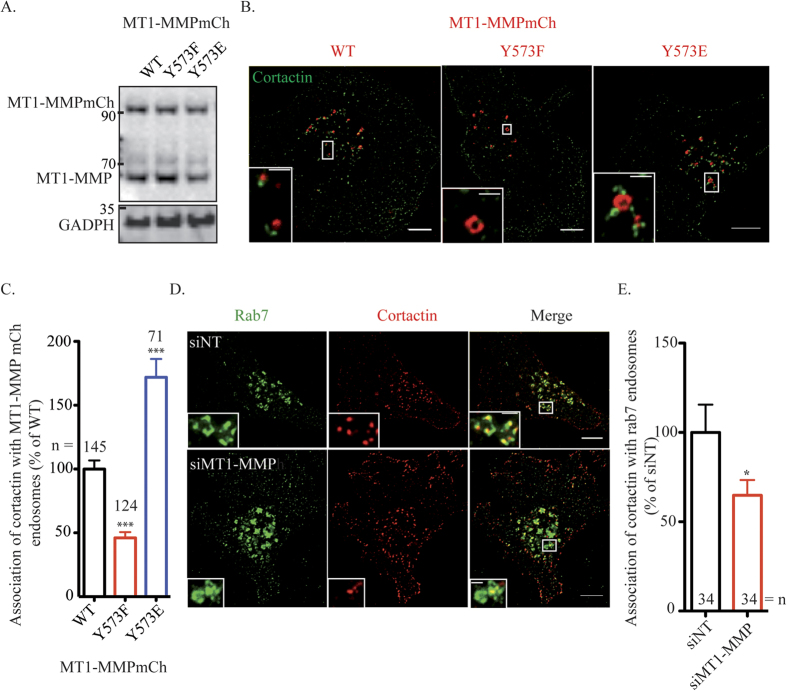
Tyrosine phosphorylation of MT1-MMP control cortactin recruitment on MT1-MMP endosomes. **(A)** Expression of MT1-MMPmCh mutants in MDA-MB-231 cells **(B)** MT1-MMPmCh WT, Y573F and Y573E were overexpressed in MDA-MB-231 cells during 24 hours. Cells were plated on gelatin and immunolabelled with antibody against cortactin (in green). Z-projection. Scale bars, 5 μm (entire cell); 1 μm (boxed region at higher magnification). **(C)** Quantification of cortactin on MT1-MMPmCh endosomes in MDA-MD-231 cells. The y-axis indicates mean cortactin intensity associated with MT1-MMPmCh-containing endosomes normalized to the value in control MT1-MMPmCh WT cells (in percentage) ± SEM (n is the number of cells analyzed, from at least 3 independent experiments). ***P < 0.001. **(D)** MDA-MB-231 cells were treated with indicated siRNAs (siNT, for non-targeting siRNA), plated on gelatin and stained with antibodies against cortactin (in red) and Rab7 (in green). Z-projection. Scale bars, 5 μm (entire cell); 1 μm (boxed region at higher magnification). **(E)** Quantification of cortactin on Rab7 vesicles in MDA-MD-231 cells. The y-axis indicates mean cortactin intensity associated with Rab7-containing endosomes normalized to the value in control siNt-treated cells (in percentage) ± SEM (n is the number of cells analyzed, from at least 3 independent experiments). *P < 0.05.

WT, hypo- (Y573F) or hyper-phosphomimetic (Y573E) variants of MT1-MMPmCh were overexpressed to similar levels in MDA-MB-231 cells and accumulated in cytoplasmic vesicles that we previously identified as late endosomes/lysosomes (Fig. 2A,B)^19^,^21^,^22^. A hallmark of MT1-MMP-positive endosomes is the presence of F-actin-, cortactin-rich puncta, which depends on WASH and Arp2/3 complex for their formation and are required for MT1-MMP surface delivery^22^. Interestingly, as compared to MT1-MMP WT-containing endosomes, we observed opposite ∼30% decrease and ∼80% increase of cortactin association to MT1-MMP Y573F- or Y573E-containing endosomes, respectively (Fig. 2B,C). In addition, MT1-MMP knockdown leads to a significant ∼40% reduction in cortactin-positive puncta associated with Rab7-positive late endosomes (Fig. 2D,E).

**Figure 3:**
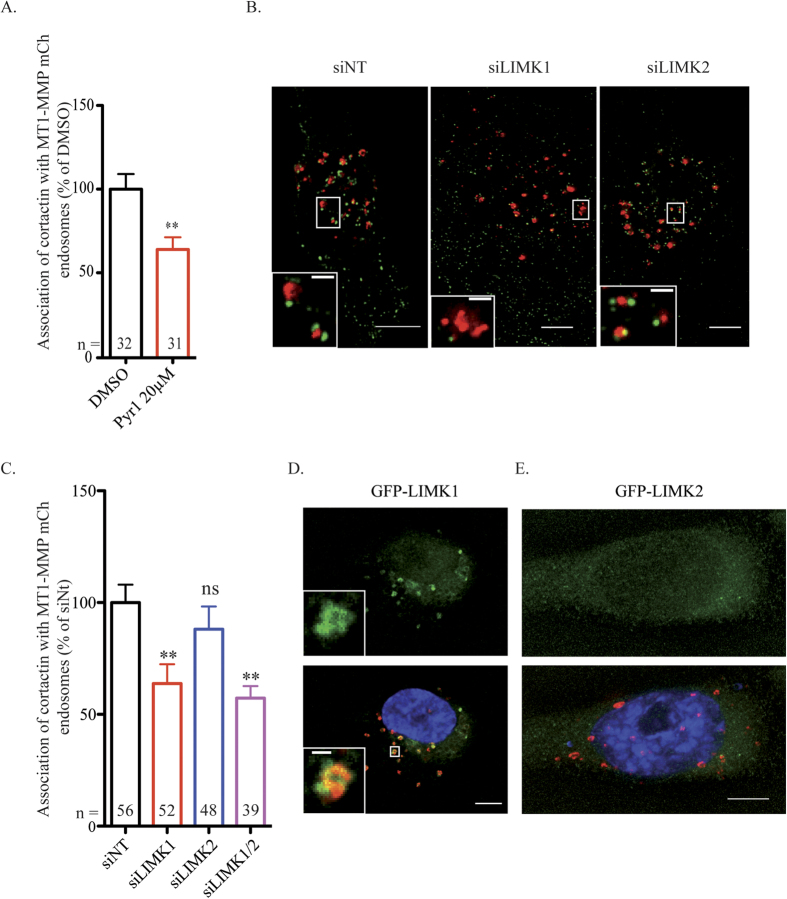
LIMK1 control cortactin recruitment on MT1-MMP endosomes. **(A)** Quantification of cortactin on MT1-MMPmCh-containing endosomes. Y-axis indicates mean cortactin intensity associated with MT1-MMP endosomes normalized to the value in control DMSO-treated cells (in percentage) ± SEM (n is the number of cells analyzed, from at least 3 independent experiments). ***P < 0.001. **(B)** MDA-MB-231 cells stably expressing MT1-MMPmCh were treated with the indicated siRNAs (50 μM), plated on gelatin and stained for cortactin (in green). Z-projection. Scale bars, 5 μm (entire cell); 1 μm (boxed region at higher magnification). **(C)** Quantification of cortactin on MT1-MMPmCh vesicles as in panel A. The x-axis indicates mean cortactin intensity associated with MT1-MMPmCh-containing endosomes normalized to the value in control siNt-treated cells (in percentage) ± SEM (n is the number of cells analyzed, from at least 3 independent experiments). ***P < 0.001. (**D,E)** Overexpression of LIMK1-GFP **(D)** or LIMK2-GFP **(E)** in MT1-MMPmCh overexpressing MDA-MB-231 cells. Z-projection. Scale bars, 5 μm (entire cell); 1 μm (boxed region at higher magnification).

**Figure 4:**
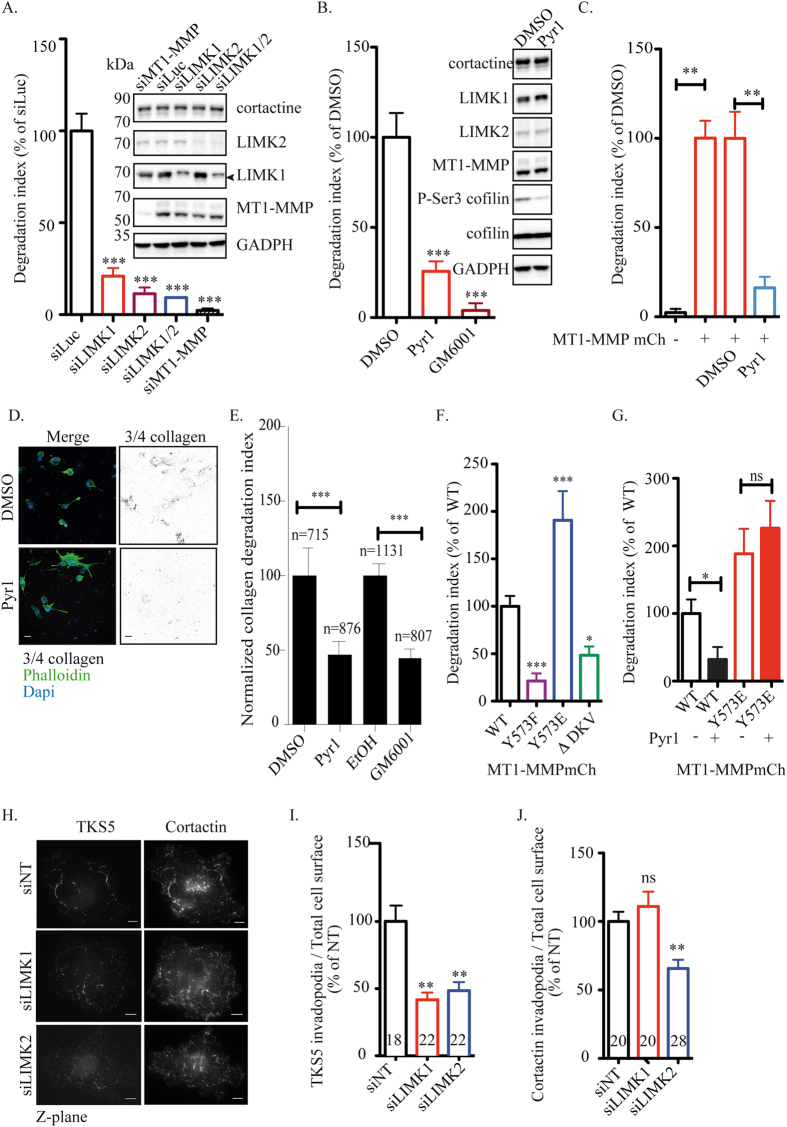
LIMK1/2 are required for MT1-MMP-dependent matrix degradation. **(A,B)** Quantification of FITC–gelatin degradation by MDA-MB-231 cells treated with indicated siRNAs (**A**) or inhibitors (**B**). Values are means ± SEM of the normalized degradation area from at least three independent experiments. (*Insets*) Immunoblotting with antibodies against LIMK1, LIMK2 and MT1-MMP of cells treated with the indicated siRNAs. Immunoblotting with antibodies against GAPDH served as a control for loading. Arrow indicates specific LIMK1 band. (**C**) Quantification of FITC–gelatin degradation by MDA-MB-231 stably expressing MT1-MMPmCh treated with indicated siRNAs (black bars). (*Insets*) Immunoblotting with indicated antibodies of cells treated with DMSO or Pyr1 20 μM. (**D**) MDA-MB-231 cells treated with Pyr1 or DMSO vehicle were embedded in collagen I. Pericellular collagenolysis was detected using anti-Col1-^3/4^C antibodies (in black in the inverted image). Nuclei were stained with DAPI and actin with Phalloidin. Bars, 20 μm. **(E)** Quantification of collagenolysis by MDA-MB-231 cells treated with the indicated inhibitors. Values are mean normalized degradation index ± SEM from at least three independent experiments. n represents the number of cells analyzed for each cell population. ***P < 0.001 (as compared with the control cells). **(F)** Quantification of FITC–gelatin degradation by MDA-MB-231 expressing mutants of MT1-MMPmCh. *P < 0.05. ***P < 0.001. **(G)** Quantification of FITC–gelatin degradation by MDA-MB-231 expressing MT1-MMPmCh WT or Y573E treated or not with Pyr1. *P < 0.05. ns, non significative. **(H)** MDA-MB-231 cells were treated with indicated siRNAs, plated on cy5-labelled type I collagen and respectively immunolabelled with an antibody against cortactin and TKS5. z-plane corresponding to the collagen fibers network. Scale bars, 5 μm. **(I)** Quantification of TKS5 present at the cell surface of MDA-MB-231 cells treated with indicated siRNAs plated on a layer of type I collagen. The y-axis indicates TKS5 area normalized on total cell area related to the value in control (in percentage) ± SEM. **(I)** Quantification of cortactin present at the cell surface of MDA-MB-231 cells treated with indicated siRNAs plated on a layer of type I collagen. The y-axis indicates TKS5 area normalized on total cell area related to control value (in percentage) ± SEM.

We further investigated whether pharmacological inhibition or silencing of LIMK could affect cortactin recruitment on MT1-MMP-positive endosomes through regulation of Y573. Treatment with Pyr1 resulted in a ∼40% decrease of cortactin association with MT1-MMP-containing endosomes (Fig. 3A). Interestingly, knockdown of LIMK1 but not LIMK2 similarly affected cortactin association with MT1-MMP endosomes (Fig. 3B,C). Consistently, we observed that GFP-LIMK1 but not GFP-LIMK2 associated with MT1-MMPmCh-containing endosomes (Fig. 3D,E). Knockdown of LIMK1 or LIMK2 did not affect the level of the other isoform (Figs S1B and 4A, inset). In addition, individual or simultaneous depletion of LIMK1 or LIMK2 or inhibition of LIMK activity did not affect MT1-MMP or cortactin protein level (Fig. 4A, inset).

### LIMK1 and LIMK2 control MT1-MMP-dependent proteolytic matrix degradation through regulation of Y573 phosphorylation

Functional interplay between MT1-MMP and LIMK1/2 was investigated in invasive MDA-MB-231 cells. Silencing of MT1-MMP strongly diminished invadopodial degradation of fluorescent gelatin (>95%, Fig. 4A)^16^,^43^. As compared to cells treated with an irrelevant siRNA (luciferase, siLuc), MDA-MB-231 cells knocked down for LIMK1, LIMK2 or both showed a ≥80% reduction of their degradative capacity (Fig. 4A and Fig. S1A,C). These results were independently confirmed in human breast BT-549 cell line (Fig. S2A). Similarly, inhibition of LIMK with Pyr1 resulted in a ∼70% decrease of gelatin degradation (Fig. 4B and Fig. S1D), almost as efficiently as inhibition caused by GM6001 MT1-MMP inhibitor. Moreover, inhibition of LIMK also inhibited gelatin degradation by overexpressed MT1-MMP (Fig. 4C), indicating that LIMK activity is required for MT1-MMP-mediated matrix preoteolysis. The contribution of LIMK to MT1-MMP-dependent type I collagen proteolysis was also assessed in a 3D environment. MDA-MB-231 cells were imbedded in type I collagen gel and treated overnight with Pyr1 or GM6001 inhibitors. Pericellular collagenolysis was detected using anti-Col1-^3/4^C antibodies, which detect cleaved collagen. A 50% decrease of collagen degradation was observed when MT1-MMP or LIMK was inhibited (Fig. 4D,E). All together, these data indicate that LIMK1 and LIMK2 are required for matrix proteolysis by breast tumor cells through control of MT1-MMP function.

Next, we investigated the contribution of LIMK-dependent Y573 phosphorylation to matrix degradation by MT1-MMP. Overexpression of hypophospho-mimetic Y573F mutant lead to ∼80% reduction of degradation activity (Fig. 4F). Similar results were obtained in BT-549 cells (Fig. S2B,C). Deletion of the carboxy-terminal DKV motif had a similar albeit lower inhibitory effect (Fig. 4F and Fig. S3A,B). This was in contrast to phospho-mimetic Y573E variant, which stimulated gelatin degradation by ∼1.8 fold as compared to WT MT1-MMP (Fig. 4F and Fig. S3C). Note that these mutants were expressed to similar levels (Fig. 2A). Importantly, stimulation of gelatin degradation by Y573E was insensitive to Pyr1 indicating that LIMK acts upstream of MT1-MMP to promote matrix degradation by regulating phosphorylation of Y573 (Fig. 4G).

Finally, we addressed the consequence of LIMK inhibition on invadopodia formation. Cells plated on fibrillar collagen formed linear invadopodia structures in association with collagen fibers, which could be stained with antibodies to Tks5 or cortactin invadopodia markers (Fig. 4H and Fig. S3D)^22^,^27^,^49^. Quantification of Tks5 signal in association with linear invadopodia revealed that treatment with 20 μM Pyr1 triggered a ∼60% decrease of Tks5 association (Fig. S3E). In addition, both LIMK1 and LIMK2 were required for invadopodia formation on fibrillar collagen networks (Fig. 4H,I). Interestingly, knockdown of LIMK2 but not LIMK1 affected cortactin association to linear invadopodia (∼40% decrease, Fig. 4J). Next, we investigated the contribution of LIMK-dependent Y573 phosphorylation to linear invadopodia formation. First, we observed that overexpression of WT MT1-MMPmCh triggered an increase of Tks5 association to linear invadopodia compared to parental MDA-MB-231 cells (Fig. S3F). Overexpression of hypophospho-mimetic Y573F mutant lead to ∼30% reduction of Tks5 association positive linear invadopodia formation (Fig. S3F). Deletion of the carboxy-terminal DKV motif had a stronger inhibitory effect. This was in contrast to phospho-mimetic Y573E, which stimulated invadopodia formation as compared to MT1-MMPmCh WT (30% increase, Fig. S3F).

### LIMK control MT1-MMP-dependent tumor-cell invasion in 3D type I collagen

**Figure 5:**
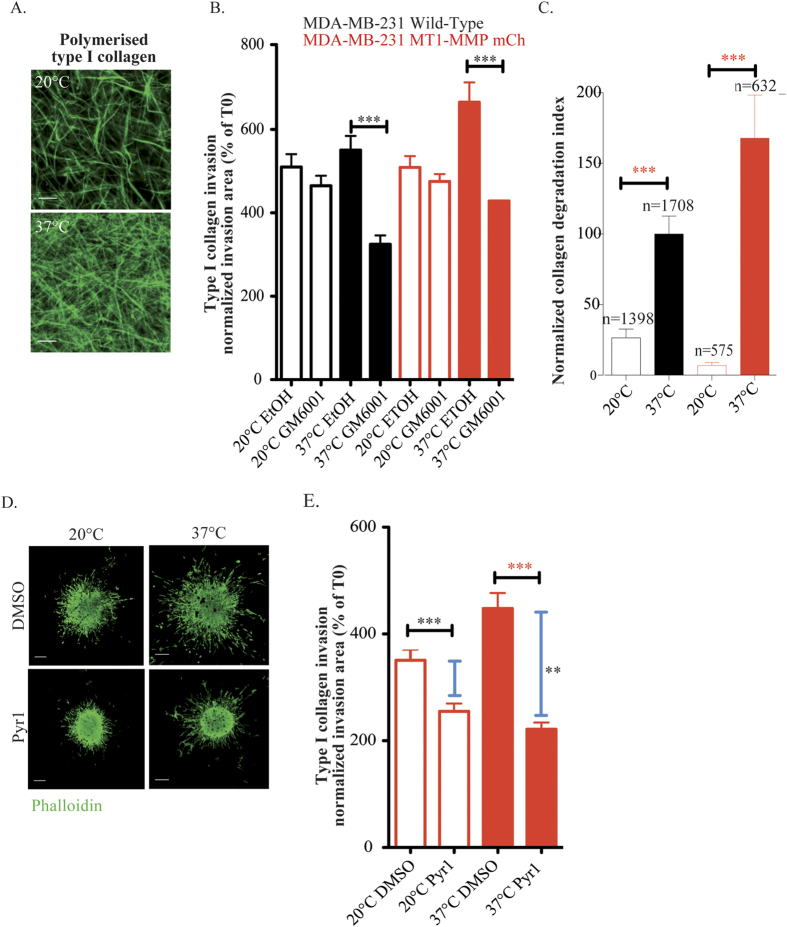
LIMK1/2 are required for MT1-MMP-dependent tumor-cell invasion. **(A)** Confocal images of cy5 labeled type I collagen polymerized at 20 °C or 37 °C. Scale bar, 20 μM. **(B)** Multicellular spheroids of MDA-MB-231 cells expressing or MT1-MMPmCh treated with GM6001 20 μM or ethanol were embedded in 3D acid-extracted type I collagen polymerized at 20 °C or 37 °C (T0) and further incubated for 2 days (T2). Data are mean invasion area in type I collagen at T2 normalized to the mean invasion area at T0 ± SEM (n = 3 independent experiments, more than 20 spheroids were analyzed for each cell population). ***P < 0.001. **(C)** Quantification of collagenolysis by MDA-MB-231 cells expressing or not MT1-MMPmCh in type I collagen polymerized at 20 °C or 37 °C. Values are mean normalized degradation index ± SEM from at least three independent experiments. “n” represents the number of cells analyzed for each cell population. ***P < 0.001 (as compared with the control cells). **(D)** Multicellular spheroids of MDA-MB-231 cells treated with Pyr1 10 μM or DMSO were embedded in 3D acid-extracted type I collagen polymerized at 20 °C or 37 °C (T0) and further incubated for 2 days (T2). Images show phalloidin-labelled spheroids collected at T2. Scale bars, 200 μm. **(E)** Data are mean invasion area in type I collagen at T2 normalized to the mean invasion area at T0 ± SEM (n = 3 independent experiments, more than 20 spheroids were analyzed for each cell population). ***P < 0.001. **P < 0.01.

We made use of the differential permissivity of large *vs.* small pore size collagen networks to invasive migration to investigate a LIMK contribution to the invasive potential of breast tumor cells in a 3D environment. Large pore size collagen gels obtained by polymerization at 20 °C are permissive to invasion, while small pore size collagen networks polymerized at 37 °C require MT1-MMP activity for invasion^50^ (and see Fig. 5A). Confirming previous observations^50^, inhibition of MT1-MMP activity by GM6001 impaired invasion of MDA-MB-231 cells overexpressing or not MT1-MMPmCh in non-permissive type I collagen gel polymerized at 37 °C (Fig. 5B). In contrast, invasive potential was not affected upon MT1-MMP inhibition in cells invading through large pore size collagen matrix polymerized at 20 °C. Interestingly, we observed that the extent of collagen degradation was much larger when cells were embedded in small pore size as compared to large pore size collagen gels (Fig. 5C). In addition, overexpression of MT1-MMP correlated with an increase in invasive and degradative potential of the cells only in small pore size (37 °C) collagen environment (Fig. 5B,C). Inhibition of LIMK activity by Pyr1 resulted in a ∼25% reduction of invasion in large pore size type I collagen, while a significantly larger (∼50%) inhibition was observed in small pore size collagen matrix (Fig. 5D,E). This body of results highlights a requirement for LIMK for both MT1-MMP-dependent matrix degradation and invasion in 3D type I collagen environment.

## Discussion

Here, we show that MT1-MMP interacts with both LIMK1 and LIMK2. MT1-MMP is a key metalloproteinase regulating matrix degradation^4^,^5^,^6^,^8^,^9^,^51^,^52^. We found that single knockdown of LIMK1 or LIMK2 triggered a strong decrease (about 80%) in matrix degradation without affecting MT1-MMP levels or expression of the other LIMK isoform in MDA-MB-231 cells and BT-549 breast cancer cells. In contrast to the well-known LIMK substrate cofilin, which is phosphorylated on S3 residue^29^, we identified Y573 in the cytoplasmic tail of MT1-MMP as a new substrate of dual-specificity LIMKs (Fig. 1). We further show that phosphorylation of Y573 of MT1-MMP by LIMK is required for its function in matrix degradation (Fig. 4G). Then, we further sought to differentiate the contribution of LIMK on tumor cell invasion due to the control of MT1-MMP function from its known regulatory role on actin and microtubule cytoskeleton dynamics^29^,^48^. We took advantage of the finding that MT1-MMP dependency of tumor cells during invasion in a 3D collagen network is influenced by matrix porosity, *i.e.* large pore size collagen gel is permissive for invasive migration in the absence of MT1-MMP activity, while invasion through small pore size collagen requires pericellular collagenolysis by MT1-MMP^50^. Our data indicates a contribution of LIMKs to MT1-MMP-independent invasion in large pore size collagen gel in agreement with their regulatory role on actin and microtubule cytoskeleton dynamics^29^,^48^. Our data also clearly demonstrates that LIMKs are required for MT1-MMP-dependent matrix degradation and cell invasion in dense fibrillar type I collagen networks. This is associated with LIMK1/2 requirement for the formation of linear invadopodia on collagen networks. Pericellular collagenolysis by MDA-MB-231 cells invading through small pore size collagen gel is visible, which requires MT1-MMP and LIMK activity. Interestingly, a previous study reported that LIMKs were required for invasive path generation by tumor cells and carcinoma-associated fibroblasts using another small LIMK inhibitor (LIMKi)^38^. Our findings strongly suggest that LIMK affects invasive tumor cell migration by controlling MT1-MMP function and pericellular collagenolysis activity through direct phosphorylation of Y573 in MT1-MMP cytoplasmic tail.

We show that depletion of LIMK1 but not LIMK2, or inhibition of LIMK activity by Pyr1 diminishes cortactin patches on MT1-MMP endosomes (Fig. 3B). Similarly, phosphorylation of Y573 of MT1-MMP is required for the recruitment and/or stabilization of cortactin on MT1-MMP-positive endosomes (Fig. 2B,C). These results suggest that tyrosine phosphorylation of MT1-MMP is required for recruitment and/or stabilization of cortactin on MT1-MMP-positive endosomes. We recently reported that inhibition of atypical PKC in MDA-MB-231 cells increases cortactin patches on MT1-MMP-positive endosomes and correlates with defects in matrix degradation and invasion in 3D collagen^19^. Inhibition of atypical PKC also correlates with cortactin phosphorylation defects and increases endosomal F-actin/cortactin accumulation^19^. As a result, inhibition of atypical PKC decreases matrix degradation and invasion potential of MDA-MB-231 cells. This situation contrasts with the effect of overexpression of Y573E phosphomimetic mutant of MT1-MMP, which increases matrix degradation (Fig. 4F), while it promotes cortactin accumulation on MT1-MMP-positive endosomes (Fig. 2B,C). All together, these data suggest that a proper balance of endosomal cortactin association is required for MT1-MMP transport in the endosomal system. We hypothesize that LIMK1-mediated phosphorylation of MT1-MMP Y573 residue (or overexpression of MT1-MMP/Y573E variant) stimulates endosomal actin assembly and whereby promotes MT1-MMP recycling to plasma membrane invadopodia^22^,^27^. Oncogenic Src kinase, which phosphorylates MT1-MMP Y573^40^ may similarly regulate MT1-MMP delivery to the surface by controlling endosomal cortactin/actin pools. Of note, a L571L572Y573 motif in the cytosolic tail of MT1-MMP is known to interact with F-actin in invadopodia^27^, suggesting that MT1-MMP may similarly influence recruitment and/or stabilization of F-actin/cortactin on late endosomes in a process that is regulated by phosphorylation of Y573. Overexpression of MT1-MMP confers invasive potential to non-invasive, MT1-MMP-negative COS cells^6^,^53^. Contrasting with a proposed role for the carboxy-terminal cytoplasmic domain of MT1-MMP in MDA-MB-231 cell invasion, MT1-MMP-dependent invasion of COS cells does not require the cytosolic domain^6^,^53^. Although the reason for this discrepancy is not clear, it may suggest that MT1-MMP trafficking routes and/or transport machineries differ in invasive *vs.* non-invasive cells. Of note, interaction of the related carboxy-terminal “EWV” motif of MT5-MMP with the PDZ domain of Mint3 regulates recycling of MT5-MMP to the cell surface^54^, suggesting a role for the cytosolic domain in the trafficking of MT-MMPs.

Our results suggest non-redundant roles for LIMK1 and LIMK2 in matrix degradation and invadopodial recruitment of Tks5 in breast tumor cells. Although both knockdown of LIMK1 and LIMK2 inhibit matrix degradation, depletion of LIMK1 specifically affects cortactin association on MT1-MMP-positive endosomes (Fig. 3B), while LIMK2 knockdown specifically affects the invadopodial cortactin pool (Fig. 4H,J). Consistent with these differential roles of LIMK1 and LIMK2, only LIMK1 localizes on MT1-MMP-positive endosomes (Fig. 3D). It is possible that the differential effects of LIMK1 and LIMK2 on endosomal and invadopodial actin/cortactin pools, respectively, involve phosphorylation of cofilin by LIMK^34^. Interestingly, silencing of LIMK1 and LIMK2 similarly affected invadopodial recruitment of Tks5 (Fig. 4H,I), suggesting that the functions of LIMK1 and LIMK2 on two distinct actin/cortactin pools influence Tks5 recruitment and/or stabilization at invadopodia by controlling MT1-MMP trafficking and localization. Along this line, MT1-MMPmCh overexpression increases Tks5 association to invadopodia and this effect is affected by mutation of Y573 residue (Fig. S3F).

Future studies should help identifying determinants of LIMK1 and LIMK2 association with distinct cellular compartments, *i.e.* endosomes and plasma membrane possibly underlying some differential functions of the two related LIMK-family members and spatio-temporal regulation of MT1-MMP phosphorylation by LIMK1 and/or LIMK2 during tumor cell invasion. Taking into account the fact that MT1-MMP and LIMK1/2 are overexpressed in cancers, in particular breast cancers, our study provides novel insights at the mechanisms regulating tumor cell invasion in a 3D context and suggest new possibilities for therapeutic interventions.

## Additional Information

**How to cite this article**: Lagoutte, E. *et al.* LIMK regulates tumor-cell invasion and matrix degradation through tyrosine phosphorylation of MT1-MMP. *Sci. Rep.* **6**, 24925; doi: 10.1038/srep24925 (2016).

## Supplementary Material

Supplementary Information
