# Supplementary material for: LIMK Regulates Tumor-Cell Invasion and Matrix Degradation Through Tyrosine Phosphorylation of MT1-MMP
Source: Sci Rep. 2016 Apr 27;6:24925. doi: 10.1038/srep24925 (PMC4847008; doi:10.1038/srep24925)
Supplement: Supplementary Information [file srep24925-s1.pdf]

Supplementary information

LIMK regulates tumor-cell invasion and matrix degradation through tyrosine phosphorylation of MT1-MMP

Emilie Lagoutte[1], Clémentine Villeneuve[1], Laurence Lafanechère [2], Claire Wells[3], Gareth E. Jones[4], Philippe Chavrier[1 *#], Carine Rossé[1 *#]

[1] Institut Curie, PSL Research University, CNRS UMR 144, Membrane and Cytoskeleton Dynamics, 75248 cedex 05, Paris, France

[2] Univ. Grenoble Alpes, INSERM U823, Institut Albert Bonniot, CRI, Team 3 "Polarity, Development and Cancer", F-38000 Grenoble France

[3] Division of Cancer Studies, King's College London, London, United Kingdom

[4] Randall Division of Cell and Molecular Biophysics, King's College London, London, United Kingdom

[#] Co-last authors

[*] Corresponding authors (carine.rosse@curie.fr; philippe.chavrier@curie.fr)

Running title: LIMK1/2 is a tyrosine kinase for MT1-MMP

Keywords: LIM kinase, cell invasion, MT1-MMP, tyrosine phosphorylation, matrix degradation

Legends

Supplemental figure 1. Effect of individual siRNAs against LIMK1 and LIMK2 on matrix degradation in MDA-MB-231 cells. (A) MDA-MB-231 cells treated with siRNAs against luciferase (siLuc) or siLIMK1 or siLIMK2 were plated for 5 h on fluorescently labeled cross-linked gelatin and then analysed by labeling for cortactin and F-actin. Gelatin degradation was focal and coincided with cortactin-, F-actin positive invadopodia forming at the adherent plasma membrane. Invadopodia formation and gelatin degradation were impaired in -depleted cells. (Scale bar: 5µM). **(B)** Immunoblotting with antibodies against LIMK1 and LIMK2 of cells treated with the indicated siRNAs. Immunoblotting with antibodies against GADPH served as a control for loading. **(C)** Immunoblotting with antibodies against LIMK1, LIMK2 and cortactin of cells treated with the indicated siRNAs. **(D)** MDA-MB-231 cells treated with DMSO (control) or Pyr1 (20µM) were plated for 5 h on fluorescently labeled cross-linked gelatin and then analysed by labeling for cortactin and F-actin. Gelatin degradation was focal and coincided with cortactin-, F-actin positive invadopodia forming at the adherent plasma membrane. Invadopodia formation and gelatin degradation were impaired in -depleted cells. (Scale bar: 5µM).

Supplemental figure 2. Effect of LIMK1 and/or LIMK2 depletion on matrix degradation in BT-549 cells. (A) Quantification of FITC–gelatin degradation by BT-549 cells treated with indicated siRNAs. Values are means ± SEM of the normalized degradation area from at least three independent experiments. ***, $P<0.001$, **, $P<0.01$. (*Insets*) Immunoblotting with antibodies against LIMK1 or LIMK2 of cells treated with the indicated siRNAs. Immunoblotting with antibodies against GAPDH served as a control for loading. **(B)**

Quantification of FITC–gelatin degradation by BT-549 cells expressing mutants of MT1-MMP-mCherry. *, $P<0.05$. **(C)** Expression of MT1-MMP mCh mutants in BT-549 cells.

Supplemental figure 3. Effects of MT1-MMPmCh mutants expression on matrix degradation and invadopodia formation in MDA-MB-231 cells. (A&C) MDA-MB-231 cells expressing mutants of MT1-MMP-mCherry were plated for 5 h on fluorescently labeled cross-linked gelatin and then analysed. Gelatin degradation was focal at the adherent plasma membrane. (Scale bar: 5µM). **(B)** Expression of MT1-MMP mCh ΔDKV mutants in MDA-MB-231cells. **(D)** Immunoblotting with antibodies against LIMK1, LIMK2 and cortactin of cells treated with the indicated siRNAs. **(E)** Quantification of TKS5 present at the cell surface of MDA-MB-231 cells treated with DMSO or Pyr-1 (20µM) plated on a layer of type I collagen. The y-axis indicates TKS5 area normalized on total cell area related to the value in control (in percentage) ± SEM. **(F)** Quantification of TKS5 present at the cell surface of MDA-MB-231 cells expressing mutants of MT1-MMP-mCherry or not (control) plated on a layer of type I collagen. The y-axis indicates TKS5 area normalized on total cell area related to the value in control (in percentage) ± SEM.

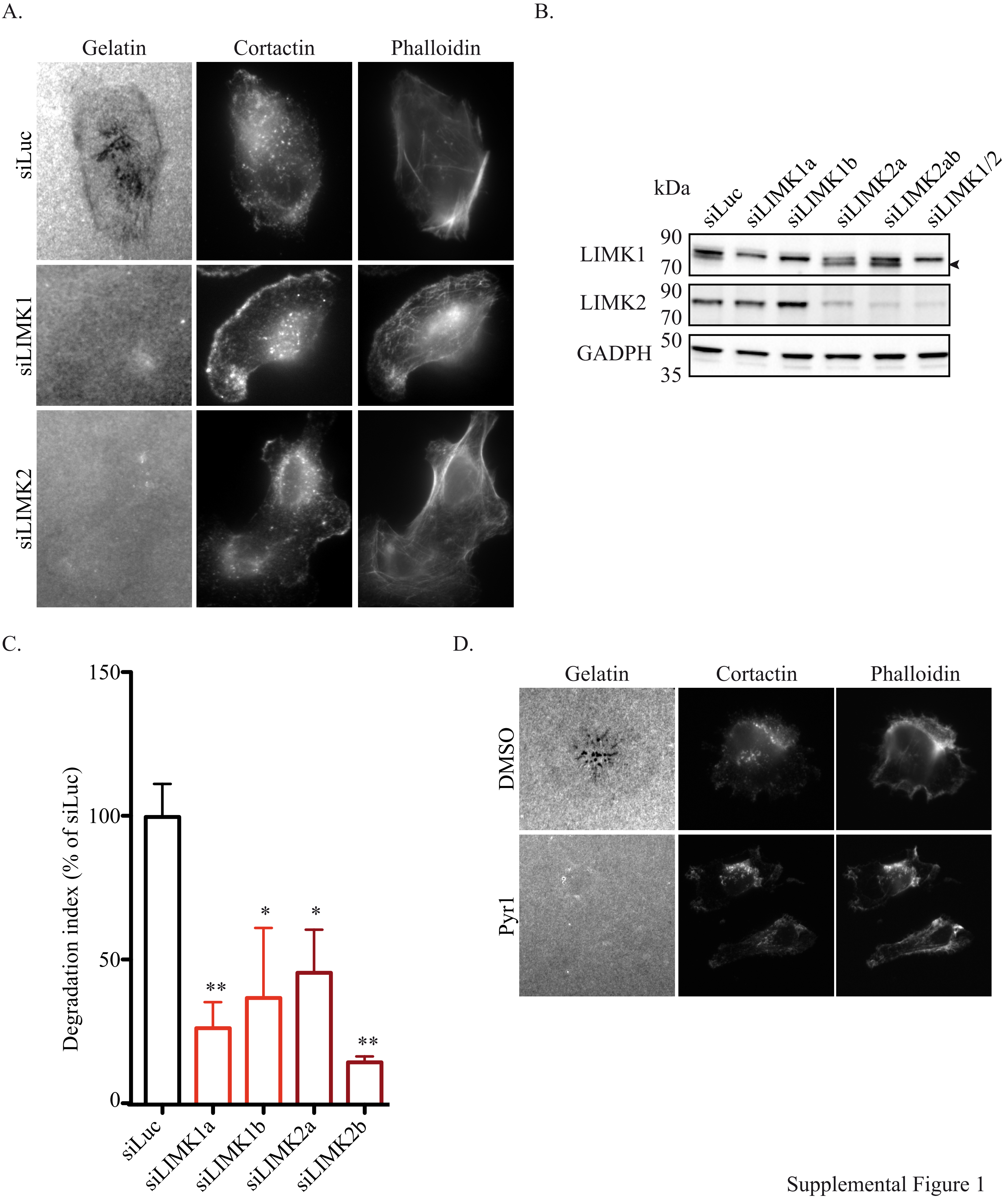

Supplemental Figure 1

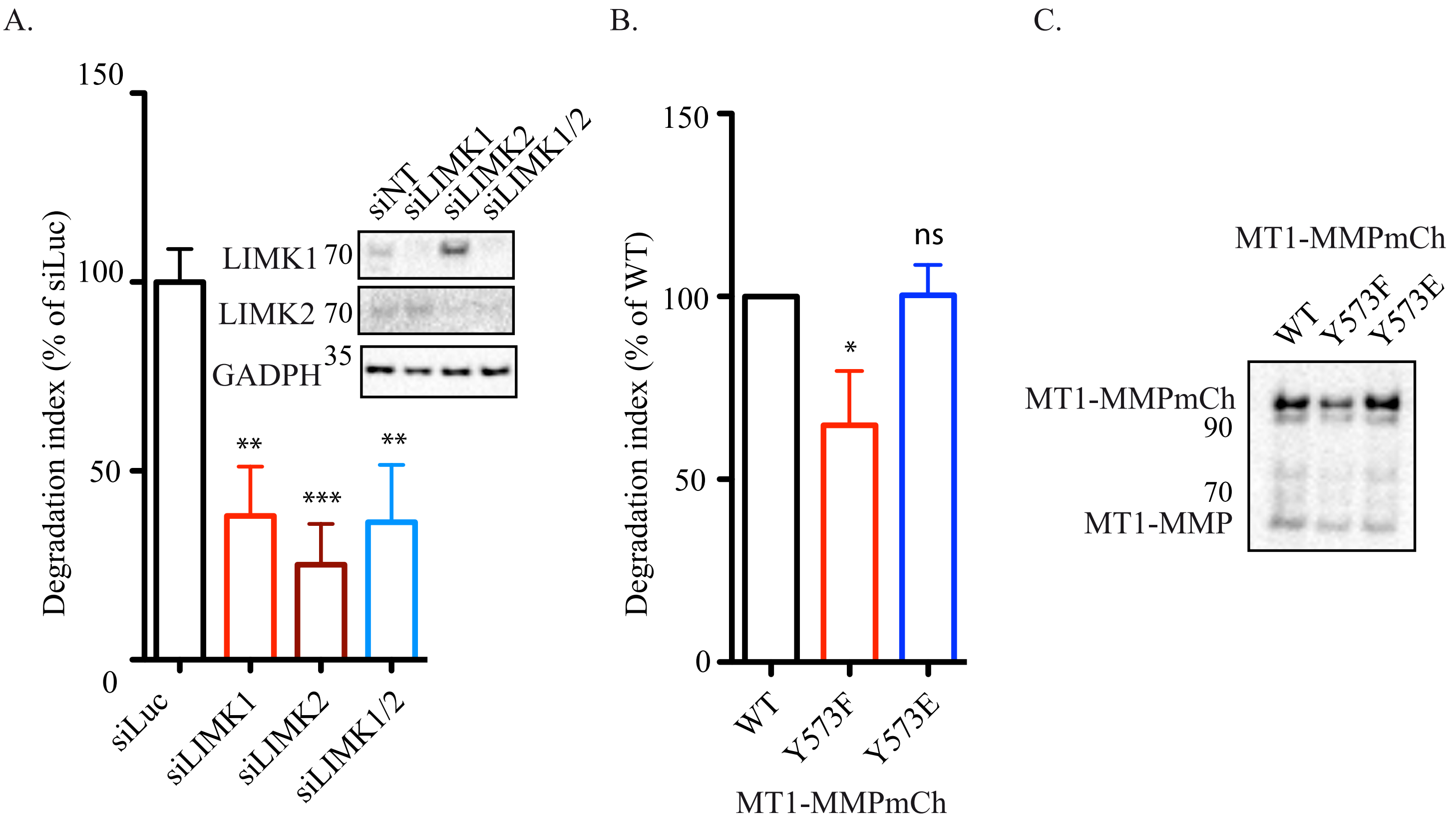

Supplemental Figure 2

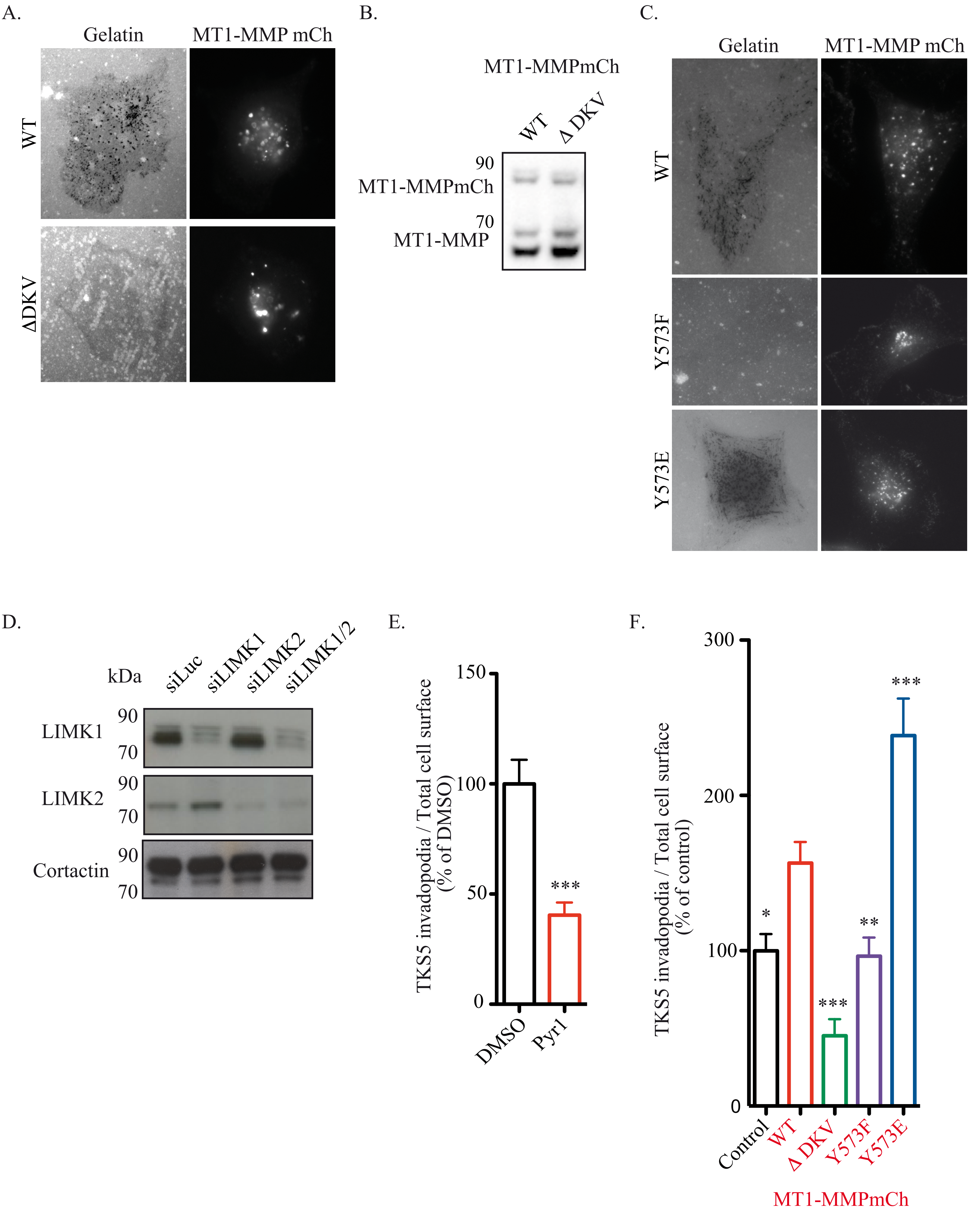

Supplemental Figure 3
